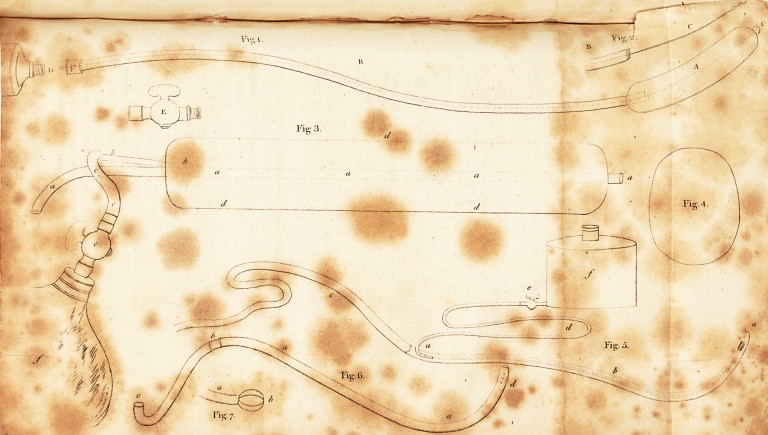# Mr. Arnott on Obstructions in the Urethra, Rectum, &c.

**Published:** 1821-03-01

**Authors:** 


*Cases illustrative of the Treatment of Obstructions in the Urethra, &c. by the New Instrument, the Dilator; with further Directions, to facilitate its General Adoption: also, a Case of the Extraction of Stone from the Male Bladder without cutting it, by the Dilator; with an account of Improvements of the Method of Dissolving Stone by Injection, and of the common Operations of Lithotomy.* By James Arnott, Member of the Royal College of Surgeons in London. 1 vol. 8vo. pp. 119. London, 1821, with a plate.

In one of the numbers of our *Quarterly Series*, we gave some account of Mr. Arnott's dilator; and we have heard favourable reports of it since that period, both in this country, and on the Continent. In the present publication, our author delineates certain improvements in the instrument itself, gives minute directions for using it, and recommends an extension of its application.*

Our author remarks that the process of destroying stricture, in the urethra, by means of caustic, is now totally relinquished, " from the experienced difficulty of confining its action to the stricture alone; and from the severe pain, irritation, and danger occasioned when this is not accomplished." *Dilatation* is, therefore, the remedial measure universally resorted to; but to the *action* of the instruments commonly in

---

* These instruments may be procured of Mr. Ironside, No. 7, Philpot-lane, Fenchurch-street.

use, Mr. Arnott objects, as being that of the wedge—"and thence they have four radical defects."

"The first of these which I shall notice, and which proceeds from the necessity of pushing forward the instrument when it is at, or in, the stricture, to dilate it, is, that the urethra is often pierced by it before or behind the stricture, causing hæmorrhage, false passage, and urinary abscess; or, which is productive of similar effects, the stricture is torn from its situation, and carried forward on the instrument. These misfortunes are particularly liable to occur when much force is employed to dilate, either for the purpose of opening a common stricture very quickly, or when a hard stricture will not yield at all, to milder measures. The second defect is, that, from this progressive motion being necessary to the dilatation, much needless pain and irritation are produced by the friction of the instrument upon the tender parts as it advances. A third great defect of these instruments, from their being unchangeable in dimension, is, that, as the orifice of the urethra is of smaller diameter than the rest of the canal in its healthy condition, it cannot readily, or without much irritation, admit an instrument of sufficient size to dilate a stricture behind, to the level of the canal there; and as stricture not sufficiently dilated, commonly returns on intermitting the process of distension, a permanent cure is thus not obtained. The fourth defect is, that such an unchangeable instrument cannot act equally on the whole of a long stricture, or on several co-existent, at once." P. 3.

It appears to us that the above objections to bougies, &c. are by no means imaginary, whether the dilator be free from them or not. Mr. Arnott thinks it is free from them all; and for the following reasons.

"It consists of a strong, air-tight membranous tube, as of oiled silk lined with thin gut, about an inch and a half in length, which is introduced into the stricture in its empty or collapsed state, and is then filled to the necessary degree of pressure, with air or water, from a syringe without; and is again emptied before being withdrawn. The dilator, while opening the stricture, remains precisely in the same position within it; so that however strongly its action may be required or exerted, even when an old stricture is completely opened by it at one application, it cannot possibly, like bougies, either pierce the urethra, or tear it. As it is introduced in its shrunk or collapsed state, no painful or injurious friction is then occasioned. And, from its being changeable in dimensions, it will enter an urethra with the narrowest orifice, and still dilate a contraction in any part, to the natural size, or beyond that, if necessary, without stretching, like the bougie, the whole canal anterior to it. For the same reason the dilator acts equally on the whole of a long stricture, or on several strictures at once." 4.

Mr. Arnott next enters into a particular examination of the advantages and disadvantages above enumerated; for the details of which we must refer to the volume itself. But the author having kindly lent us the plate to work off impressions enough for this number of the Journal, we shall here give a slight explanation of the figures which may possibly enable those of our brethren in foreign countries and the colonies, who have no means of procuring the work or the instruments, to construct them themselves.

"Fig. 1. represents, in its distended state, a common urethra dilator of size No. 14. The part which acts upon the stricture is the short tube of strong silk, A, when distended. This is lined with thin gut, to make it air-tight, and covered with the same, or with varnish, to make it smooth for passing down. One end of it is tied upon the extremity of the directing wire C, and the other upon the extremity of the tube, or cannula, B. The wire C, which runs through, and projects beyond the cannula B, serves to conduct the dilating tube in its collapsed state into the stricture, and by the cannula, the distending fluid, air or water, is injected from the syringe D. The stop cock E, screwed into the outer end of the cannula, at F, retains this injected fluid.

"Fig. 2 represents the skeleton of part of the dilator, without the distensible tube, that its construction may be better understood.

"The cannula B may be of the common elastic catheter tube, or of tin, which is flexible, or of silver. To its outer end at F, is fixed, by cement, a small connecting piece of brass, to receive the corresponding screw end of the stop cock E, or of the syringe D, when only momentary distension is made, and the cock therefore is not required. At its other end (fig. 2,) it is represented as roughened, that the silk and gut tube may be more securely attached to it.

"The wire C, (which is represented by the dotted line in fig. 1.) is of silver, prepared so as to be elastic, as small as the necessary degree of strength will permit, and sufficiently long to project from both ends of the cannula; from the inner end, as much as the length of the distensible tube is required to be, and about one-fourth of an inch at the outer end, where it has a knob on it, or hook, to prevent the possibility of its slipping from the tube, and being left in the urethra. At its point (fig. 2,) there are two knobs or risings, between which the silk is tied on; one constitutes the point of the finished instrument, and is one-tenth of an inch in thickness, that it may pass easily; the other, a quarter of an inch distant from it, is merely large enough to prevent the tying from slipping back upon the wire. The wire is freely moveable to and fro in the conducting tube, for several reasons, such as to facilitate the tying on of the silk, so as not to leave it twisted, and that the silk tube be neither too slack nor too tight on the wire. That the surgeon may be able, however, to direct the point of the wire to the opening of the stricture, the whole should receive the double or S curve, natural to the urethra, as is shewn in the plate." 23.

The syringe D is a brass forcing syringe, 3½ inches long, and half an inch in internal diameter.

"For ordinary cases of stricture, the distensible tube may be constructed of strong silk ribbon, with the edges sewed together, and having its seam turned inwards, lined and covered with thin gut. Such a tube will in every instance be found of sufficient strength to bear the requisite degree of pressure. The greatest pressure of the thumb upon the piston of such a syringe as has been described, will rarely rupture it, and this force is more than the hardest stricture can for a moment resist. When little bulk in the dilator is desirable, as in the very beginning of the treatment, the thinnest oiled silk, lined with a gut, will be preferable; but as this will often give way to great pressure, it is proper previously to ascertain how much it can resist, and to point this out by a mark or check on the piston rod of the syringe. Besides the loss of the distensible tube from such an occurrence, there is a chance of injury to the urethra from the lining gut then protruding forcibly through the breach in the silk. In some very narrow strictures I have even used at first, merely a bit of single or double gut without a covering of silk at all; but besides the want of strength, in such a tube, to bear any useful degree of pressure for hard strictures, it soon enlarges from the moisture, and is thus apt to distend the sound as well as the contracted parts of the canal. It is possible that some kinds of gut may be naturally, or by preparation, sufficiently strong to bear momentary useful pressure, yet this is not particularly desirable, for although such a tube might be simpler, it would not endure, by any means, so long as that of silk; and a silk tube dilator, of moderate diameter, when collapsed, is as small as the smallest point that can safely be introduced through a stricture. The dimensions of the silk tube will vary of course according to the circumstances of the case in which it is to be employed. If several strictures are to be dilated at the same time, and if they are situated in the curved part of the urethra, the distensible tube must be long, and corresponding to this curve; but, on ordinary occasions, it should seldom exceed two inches in length, and then the curve is unnecessary. The regulation of the diameter of the dilator will be afterwards noticed.

"The gut which I have preferred for these purposes, is that of the cat. When prepared, by stripping off the outer fleshy coat and inner villous one, it is exceedingly thin, and yet sufficiently strong. That the gut may be completely supported by the silk tube when distended, it must be at least of equal dimensions with it; and it is well to insure this by choosing it of larger size. When the stricture will admit an instrument of considerable size, as in stricture of the rectum, in order to preserve the dilator long air-tight the lining gut may be double. When the silk has a covering of gut, which, on several accounts, answers better than varnishing it, this outer gut must be pierced in several parts, in order that any of the fluid escaping from the inner gut may have free escape into the urethra, and not distend the covering beyond the silk.

"The only part of the preparation of the dilator requiring nicety of execution, is the attachment of the distensible tube to the conducting tube and wire, which must be at once very neat and very secure. The silk tube and lining gut should be tied on together, the artist taking care that the wire be kept exactly in the axis of the tube, or that the wrinkles or folds at the extremity be equal all round. The tyings may be made conical by notching the extremity of the silk after two or three turns of the small strong waxed silk thread have been made round it, and by then continuing the thread completely over it. The tyings should then be smoothed by a coating of bougie wax, and if unvarnished silk has been used, the operation is completed by covering both the silk and the tyings with a bit of gut. The secure attachment of the distensible tube to the cannula and wire is a matter of great importance; for, should the silk become detached in the canal beyond the stricture, it might happen, that the combined action of the urethra and the flow of urine would not be able to expel it, until the stricture were fully dilated; it behoves, therefore, both the instrument-maker, and the surgeon, to be careful that there exist no such hazard. The accident would prove great negligence.

"The gut must be wet during the preparation of the instrument, and at each time of using it, to prevent its cracking, or the escape of the air under the dry and shrivelled tyings. After use, the water must be as much extracted as possible, and then it should be inflated and put aside to dry. This prevents the rotting of the gut. When the distensible tube consists of unvarnished silk, lined and covered by gut, it is more easily both dried and moistened, than when varnished silk is used." 27.

After thus describing the instrument itself, our author comes next to the mode of application. One difficulty experienced by surgeons at first is, to know when the distensible tube is exactly in the stricture. Careful previous admeasurement will in general ascertain this, or a part of the outer extremity of the tube may be enlarged, in the form of a button, which in introduction, will be stopped there, and shew the stricture.

The distensible tube being within the stricture, the syringe is to be applied, and "continued gradual pressure is best made by injecting air, the elasticity of which continues the dilatation as the stricture gives way, and yields to any momentary violent spasm of the parts; and more is afterwards injected, or part allowed to escape by the cock, according to the patient's sensations. If the dilatation is intended to be sudden and momentary, then the injection of water will, on several accounts, be preferable to air." 30.

Our author thinks that, in the greater number of strictures, *momentary and considerable* distension by the dilator is the best method of treatment, and gives less uneasiness to the patient than any other.

"The distension is exclusively confined to the hard, and often nearly insensible contraction, and the short stay of the instrument in the canal, occasions no painful spasm or irritation from the ineffectual attempts of the urethra to expel the foreign body." 31.

Nevertheless he prefers, in general, to accomplish the distension by several applications, rather than by one. "Gradual continued dilatation is generally the best plan when the stricture is of the long species." In these cases, a variety of size in the dilator, though not absolutely necessary, is more adviseable than using but one size throughout." In respect to repetition, it is best to allow the irritation arising from the preceding application to nearly subside, before the dilator is re-applied. Two or three days of interval will be the least that can be allowed. A dilator measuring, in its distended state, one-third of an inch in diameter, is, our author thinks, about the natural average of the urethra, "and if the stricture has been *quickly* distended to this extent, it will probably, in most cases, be permanently so distended." Its introduction may be gradually left off, or the patient may be instructed to use in its place a large bougie for the same period.

Here our author details eight cases of stricture of urethra, treated by the dilator, and one case of stricture of the rectum. For the particulars of the former class we must refer to the work, the principal features of the latter case we shall here insert. Mr. Arnott prefaces the case with some observations on the superiority of the dilator, in intestinal strictures, over the bougies in common use.

"The dilator, which is introduced within the stricture, and again extracted, in a soft, pliable, collapsed state, which can act equally on any length of obstruction, which may carry the dilatation to any extent without ever at the same time keeping the frequently irritable sphincter of the gut distended, is obviously far preferable to any other means that has been employed for the same purpose." 73.

In the following case the bougie could not be used, owing to the great irritation caused by its friction. A gentleman of delicate reduced habit had frequent desire to stool, at which times he voided, with pain and straining, a small quantity of fæces, of a worm-like form, and generally mixed with mucus. Distressing tenesmus followed each stool, and he was troubled with flatulence, nausea, and inappetency. About three years ago his surgeon detected a stricture about three inches up, which admitted a small bougie, but the operation of passing it was always so painful, and followed by so much irritation, that he was obliged to intermit it for weeks. The gut, to the feeling, appeared pretty regularly constricted, and no other stricture could be felt.

"I introduced a rectum dilator, measuring, when inflated, twothirds of an inch in diameter, and, comparatively, with the bougie he had formerly employed, it went very easily. I inflated it as much as the patient's feelings would allow of. After fifteen minutes, the air was allowed to escape, and the instrument was extracted." 75.

On the third visit Mr. A. introduced the dilator, but could not keep it in so long as before, in consequence of the urgent desire to stool. A watery solution of opium was thrown up the rectum. After this the dilatation went on but slowly till the ninth day, when it was retained nearly half an hour. In six weeks the contraction was removed—the bowels had greatly recovered their natural functions, and Mr. Arnott left off attendance.

STONE IN THE BLADDER.

At page 79 of the work before us, Mr. Arnott enters into a short criticism on the present methods of operating in lythotomy—gives some account of new securities against several of the dangers attending these operations—and describes a new method of injecting for the solution of stone, together with a case of stone extracted by means of the dilator.

We shall pass over our author's observations on the internal use of lithontriptics, since little is now expected from such medicines, excepting as correctives of the calculous diathesis.

The idea of dissolving stones in the bladder, by means of solvent menstrua injected into that receptacle was eagerly seized by chemists, and many of Fourcroy's experiments shew that small uric acid calculi may be softened and dissolved, in a few days, by immersion in watery solutions of alkalies so mild as to be swallowed;—while calculi composed of the earthy phosphates may be still more quickly dissolved by the nitric and muriatic acids diluted so as to be no sourer than lemonade, and hardly more acrid than the urine itself. Dr. Marcet mentions the case of a person in St. Thomas's hospital, where a lithontriptic injection, consisting of 23 drops of muriatic acid to four ounces of water, was repeatedly used, and retained for upwards of an hour, without producing the least inconvenience. "This is a quantity of acid, says Mr. Arnott, double of that which, in conducting some experiments on this subject, I found very rapidly to dissolve an earthy calculus immersed in it." Mr. Arnott thinks that, under all circumstances, it seems extraordinary that the practice of injections should have been totally relinquished. The reasons, he imagines, may be sought, first, in the exceedingly imperfect method of injecting the bladder, hitherto practised—and secondly, the difficulty of ascertaining the kind of calculus in the bladder. The latter difficulty might be got over, if the smallest particle of the stone could be procured

—and he thinks it might be procured in the following manner:—

"When the stone comes to the orifice of the bladder, let an open-pointed catheter (having of course a ball-ended wire filling it during the introduction) be passed till it touch it, and by this a small circular saw, like that of the trephine, may then be introduced to grate off from the calculus, by a few turns, a sufficient quantity of dust for examination." 85.

The method of injecting hitherto has been to throw a large quantity, at once, of the solvent into the bladder, there to remain as long as the bladder will bear it, repeating the process according to the sensibility of the parts. The great defect here is, that the solvent cannot remain in contact with the stone, in a state of purity, owing to the constant descent of urine from the kidneys, which dilutes it, or possibly renders it inert. To obviate this defect, Dr. Neil Arnott contrived an apparatus called the double catheter.

"The double catheter may be made of metal, or of elastic gum. When of metal, it is formed by running a partition along a common catheter, so as to divide it into two channels, which open near its point, by distinct holes of the usual size. By one of these channels liquid may be passing into the bladder while it is again escaping, mixed with the urine, by the other. When of elastic gum, it is formed by inserting a small catheter into a larger one, and using the first for the injection of a fluid, while the latter allows it again to run off. In either construction separate flexible tubes must be attached to the outer extremities of the divisions or catheters, as prolongations of these; one, to connect the catheter with the reservoir from which the fluid is to enter by it, the other to carry off the waste fluid and urine to a fit receptacle. This apparatus has other obvious applications in affections of the bladder, besides that of dissolving stone. It is well adapted to relieve irritable bladder, in a great variety of cases in which it occurs, by allowing the acrid urine to run off immediately on descending from the kidneys, while any bland or medicated liquid may be kept circulating in the apparatus, and occupying the bladder in the desirable quantity instead of the urine. Again, it enables us to dilate a contracted bladder; a fluid column of any height may be made to act upon the bladder for this purpose, by varying the altitude, in relation to the patient, of the reservoir and extremity of the waste pipe." 88.

The double catheter will enable us, our author thinks, to place the stone in an uninterrupted stream of its proper solvent, which, however weak it may be, will still have an effect, and there will then be no temptation to risk irritating the bladder by a solvent too strong.

Mr. Arnott has relieved irritable bladders by letting the circulation of warm water go on through the double catheter, during the sleep of the patient. With this instrument there need never be more than a few drachms of fluid in the bladder, so that the stimulus of distension will never occur,

We shall pass over a proposal which our author has made to introduce an apparatus into the bladder and round the stone, so as to admit of strong solvents being injected round the calculus. We fear the apparatus is too complicated ever to answer the purpose; at all events, we refer to the work itself for the particulars.

At page 96 Mr. Arnott commences his observations on the operation of lithotomy.

"The chief circumstances upon which the fatal terminations of lithotomy depend, are the following:—

First, Exhaustion of the powers of life from the pain of the operation.

Second, Profuse hæmorrhage.

Third, Violent inflammation.

Fourth, Protracted irritation, from an unhealthy state of the wound." 97.

The first is comparatively rare. The third circumstance, violent inflammation, is by some considered to be by far the most common occasion of death, whether arising from the violence done to the parts necessarily divided—the action of the urine on the new surface—or its insinuation and lodgment among the adjoining parts. Before stating Mr. Arnott's means of obviating these dangers, we shall introduce here some observations made on this subject, in the ninth number of the "*Quarterly Journal of Foreign Medicine*," which appear by some notes, letters, and expressions, to be obviously from the pen of Mr. Shaw, demonstrator and lecturer on anatomy in the school of Windmill Street.

The writer states that he assisted a gentleman in the country to perform lithotomy on a hale, stout man, 50 years of age. There was very little hæmorrhage during the operation, but towards the evening a slight bleeding took place. In the evening visit, the operator was persuaded by a surgeon present *to put a compress on the wound*, which was fastened by a T bandage. "In the morning the scrotum was found puffed up. In three days it was gangrenous, and the patient died, as one with effusion of urine in consequence of rupture of the urethra." The reviewer observes that the cause of this effusion is too apparent to require comment.

The next case brought forward, is that, we believe, of the late eminent actor, Mr. Rae, of one of the London Theatres.

As his case excited considerable sensation, we shall be a little particular in stating it. The patient's sufferings, prior to the operation, were excessive, and he bore the operation itself with the greatest fortitude. There was some difficulty in introducing the staff, in consequence of a stricture, "but the cutting part of the operation was done with great rapidity," by, we believe, Mr. Charles Bell. "There was very little injury done to the sides of the wound in the extraction of the stone, as it was so sandy a calculus that it was brought away in very small pieces."

"The only tedious part of the operation was syringing the bladder; and he was put to bed less exhausted by the operation than any patient we ever remember to have seen. There was very little blood lost during the operation. He begged to be allowed to lie upon his side; this was granted for a short time, as he expressed himself much relieved of pain by lying in that position. The operation was performed at half-past three: on coming to him about nine in the evening, we found him exceedingly well and cheerful, suffering very little pain. On looking at the wound, we were rather surprised to see no marks of blood or urine on the clothes; and we were, moreover, informed by the nurse and pupil in attendance, that no urine had passed; the patient had been lying on his back for the last three hours, having only lain a very short time on his side. Suspecting that some clot of blood might be the cause of stopping the wound, we passed the finger in, and were much astonished to find that it required considerable *boring* to introduce the finger into a wound, which six hours before admitted a large pair of forceps, inclosing a portion of stone, to pass. The fore finger was passed as far as the knuckle: a small quantity of blood and urine followed. It was not deemed necessary to do more, as he was not suffering, nor had he any desire to make water; and we reasonably enough imagined that, in consequence of the irritation of the kidney, produced by the operation, that little urine had been secreted. He was then given in charge to the nurse, with an urgent request that he should lie on his back." *Journ. of Foreign Med. No.* IX. *p.* 50.

Towards midnight, the patient suffered great pain in the bladder, but was relieved about six in the morning, by passing a pint of bloody water by the urethra. Throughout the three succeeding days he went on remarkably well, the urine passing freely by the wound, the patient only complaining of slight pain quite on the pubes, which was attributed to the stretching of the penis on the staff during the operation, to prevent the escape of the urine. He continued very well on the fourth day, but on the fifth he was attacked with purging and a very indistinct kind of pain in the lower region of the abdomen. The purging continued—he lost his spirits—and, though suffering no particular pain, he expressed a conviction that he should die. He died on the 13th day after the operation.

"The cause of his death was fully explained on dissection. There was a large abscess, containing portions of gangrened cellular membrane, between the pubes and peritoneum; this abscess being exactly similar to that produced by effusion of urine in other circumstances." 51.

The reviewer asks what could be the cause of this? and then endeavours to explain it thus:—

"The operation, according to the received notions, was very well performed. The stone was extracted quickly, and without any laceration of the parts, or any serious bleeding: but what followed? The wound was so little bruised by the extraction of the stone, that the healthy tumefaction of the injured parts became so great, that the whole extent of the wound was actually closed. In consequence of this, it required very considerable force to pass in the fore finger: this may appear to be a thing almost incredible, but we most solemnly avow that it was so. After seeing this, is it not easy to explain what took place? In the first case related, the obstruction was on the surface of the wound, and the urine was consequently driven into the cellular membrane, below the skin, into the scrotum; but here the obstruction was deeper, and the urine, while it was forcibly driven into the urethra (for it came with great violence), escaped by the cut in the side of the urethra, into the cellular membrane, between the bladder and pubes, and lodging there, was the cause of the abscess." 51.

A friend of the writer's stated to him that he had been present at an operation of lithotomy. Eight hours afterwards he was sent for by the friend of the patient, and found the latter suffering excessive pain in the bladder. Finding the external wound very much contracted, he forced a catheter through it into the bladder, and thus relieved the patient by drawing off a quantity of urine. There can be little doubt, the reviewer adds, that the same consequences would have followed in this case as in the last, had not the bladder been relieved.

"If to these cases, we add the well-known fact, that for the first twenty-four hours after the operation, the urine generally flows by the urethra, we shall probably come to form a correct judgment of the reason why patients die so often of effusion of urine, and knowing this, be led to the proper means of preventing it. The most obvious cause of this obstruction to the passage of the urine during the first twenty-hours, is the great swelling that takes place in the tract of the wound, and it must be evident that the less violence there is done to the parts in extracting the stone, the more healthy tumefaction will there be." 52.

We confess that we cannot see so clearly how a clean cut, and no contusion of the parts by the dragging of a stone through them, *increase* the subsequent tumefaction. We should think it was just the reverse. Are not the swelling and tension of a gun-shot wound increased (ceteris paribus) by the efforts to drag the ball out again? And is not this a fair analogy? Speaking of the means of preventing these accidents, the reviewer asks, if it be not allowable to permit the lower extremities of the patient to lie apart, instead of being closed, as they always are, and thus, in some degree, prevent the chance of effusion, either of blood or urine? If the closing of the thighs be meant to restrain hæmorrhage, he thinks that no dangerous bleeding can be thus suppressed —and an oozing should not be checked from passing outwards. Some of the older surgeons put tents into the wound; but being on an erroneous principle, the *practice*, though good, was given up when the principle was found defective. The reviewer therefore suggests the propriety of reviving the old practice, and of leaving a canula of elastic gum in the wound for the first 24 hours.

"But we must be still more astonished that the necessity of introducing a canula has not been more enforced, when we see how clear Sharp is in his observations. 'The first good symptoms after the operation, is the urine coming freely away, as we then know the lips of the bladder and the prostate are not much inflamed, for they often grow turgid, and shut up the orifice in such a manner, as not only to prevent the issue of the water, but even the introduction of the finger, or female catheter, so that sometimes we are forced to pass a catheter by the penis.' "* p. 113.

"We may now be allowed to submit, that effusion of urine is generally owing to the following causes:—

"1st. That the parts at the neck of the bladder are not cut *clean*, that they are lacerated, and the cells of the cellular membrane consequently more opened.

"2d. That in trying to push in the gorget, or from the bad management in the introduction of the forceps, a cavity is made in the cellular membrane anterior to the bladder, and thus a sac is formed for the lodgement of the urine.

---

"* We are exceedingly happy to have it in our power to give the following example of the good effects of the introduction of the canula:—

"December 8, 1820.—Henry Coleman, aged twenty-two, was cut for the stone, in the Middlesex Hospital, by Mr. Cartwright. The operation was very dexterously done, and the stone was extracted in less than three minutes after the introduction of the staff. The patient was cut at half-past twelve. At six in the evening no water had come away; a canula was then passed into the bladder. In doing this, Mr. Cartwright found the opening at the neck of the bladder very much contracted. On the canula reaching the bladder, a quantity of bloody water spurted through it, to the distance of four feet! The instrument was left in the bladder; the patient did not suffer the slightest inconvenience from it. The urine did not flow by the side of the tube, but came dribbling through it. On the succeeding day the wound appeared completely closed round the canula. On the third day the tube was removed, and *now* the opening appeared to be *larger* than on the preceding day, and the urine now dribbled freely through the wound; on the fifth and sixth day some urine passed by the penis.

"Excepting a slight pain in the bowels, combined with head-ache,

"3d. That the first incision is made too high; that there is not a depending opening for the urine to pass.

"4th. That, although every incision may be correctly made, still the passage of the urine may be obstructed by the swelling of the sides of the wound." 54.

But to return to Mr. Arnott. This gentleman, alluding to the dangerous consequences of extravasation of urine, after the lateral operation of lithotomy, proposes a syphon catheter to be kept in the urethra, which, he says, will carry off every drop of urine by the natural outlet, as soon as it descends from the kidney—"and instead of leaving a tendency in the urine to spread in the wound and adjoining parts, the action of the instrument may be made so strong as even to draw any secretion from the wound that may take place in it."

"The *syphon catheter* requires to be longer than a common catheter, and instead of one opening in the point, which is the construction of the common elastic catheter, it should have several, to diminish the chance of obstruction from the bladder touching it; and an inch of its external extremity must be turned sharply up, that it may always remain full of urine, for, should the air get into it, instead of liquid, it ceases to be a syphon, and acts only as a common catheter.* It might also be a useful precaution, to tie a bit of gut upon the outer extremity of the syphon, which would constitute a valve, allowing the escape of the urine, but by its collapse preventing the access of air. The syphon catheter must be filled with water before it is introduced, or immediately after; and it may be made to act with any desired force in removing the urine, by raising or lowering the external extremity." 103.

In speaking of the high operation, which some surgeons are endeavouring to introduce into British surgery, Mr. Arnott suggests that "a staff or strong catheter of the common form, may be made to contain, in its curved part, a sliding piece, that may be pushed out after its introduction, like the joint of a telescope; the curve would thus be lengthened to nearly a semi-circle, and of course the point might easily be felt above the pubis."—Our author conceives that, with some

---

on the third day after the operation (but which was completely removed by a purge, though leeches were applied as a preventive of inflammation), this patient has not suffered the slightest pain since the introduction of the canula, up to this, the ninth day. Some of the urine still passes by the wound, although a very large quantity passes by the penis. The whole progress of this case entitles us to say, that it may be considered as one of the most successful on record.

"We are happy to see that our opinions are strengthened by the authority of Dr. Physick, who has shewn so much ingenuity in many points of surgery. He says, that of late he has been in the constant habit of introducing a piece of canula into the wound, and since he began this practice his success has been much increased."

* Fig. 6, in the Plate.

improvement in the instruments, the high operation is likely to become the preferable one in practice.

The case of extraction of a calculus by means of the dilator, is an amplification of that mentioned in our review of Mr. Arnott's work on Stricture, in the Quarterly Series of this Journal.

We have given some account of the construction of the dilator for the use of our distant brethren; we have now only to add a few short explanations of the remaining figures in the plate given in this Number of the Journal.

"Fig. iii. is one of the Dilators used in the case detailed at p. 114, in which the Stone, *fig.* iv. was extracted from the bladder, without cutting the prostate gland.

"*a*, *a*, *a*, *a*, is a cannula, by which the urine may run off.—*d*, *d*, *d*, the distensible tube of silk, lined with gut, surrounding the former.—*b*, *b*, *b*, the small cannula, through which the distensible tube is inflated from the bag, *f*; *e*, being a stopcock to retain the air after injection, and *c*, *c*, a bit of flexible tube connecting the cock to the air tube, so that touching the cock by the syringe or bag may not jar the Dilator in the tender passage. A valve might be substituted for the cock with advantage. As it is important that this Dilator should be perfectly air tight, to prevent the necessity of withdrawing it for any other purpose than the substitution of a larger as the dilatation goes on, the silk tube should be lined with double gut.

"Fig. v. is the Double Catheter, for injecting the bladder in cases of irritation of it or contraction, and for the solution of stone: it is exhibited on a reduced scale.—*See* the general description of it, p. 87.

"*f*, is the reservoir of the liquid to be injected; *d*, the flexible tube, commencing at the stop-cock *e*, by which the liquid is conveyed to the inner catheter *a*, which then carries it into the bladder, opening at *a*; *b*, *b*, *b*, is the outer catheter, by which the fluid returns with the urine, and is directed to a proper receptacle by the flexible tube *c*, of any desired length. It is important to have the outer catheter of considerable diameter, to diminish the chance of its being obstructed by the tenacious mucus, so commonly secreted in disease of the bladder.

"Fig. vi. the Syphon Catheter, for drawing off the urine constantly and completely after the operations of Lithotomy and puncturing, or any wound of the bladder, so as to prevent the urine escaping by the wound.—*See* the description, p. 102.

"Fig. vii. is to give the idea of something made to protrude from the end of a catheter or tube, after its introduction into the bladder, which will act as a button to prevent the tube slipping out, *see* p. 103. A variety of contrivances are applicable to this purpose." iv.

We entertain strong hopes that the ingenuity displayed by Mr. Arnott in the construction and employment of the dilator, together with his general information, and professional ability, will prove equally serviceable to his brethren, and creditable to himself.

## Figures and Tables

**Fig. 1. Fig. 2. Fig. 3. Fig. 4. Fig. 5. Fig. 6. Fig. 7. f1:**